# Measures of type 2 diabetes burden in Italy assessed using the AMD dataset over a twelve year span across the Great Recession

**DOI:** 10.1038/s41598-024-54989-8

**Published:** 2024-02-28

**Authors:** Cristiana Abbafati, Luciano Nieddu, Lorenzo Monasta

**Affiliations:** 1 Department of Juridical and Economic Studies, Sapienza University of Rome, P.le A. Moro 5, 00185 Rome, Italy; 2 Department of Humanistic and International Social Sciences, UNINT University for International Studies, Via C. Colombo, 200, 00147 Rome, Italy; 3 Clinical Epidemiology and Public Health Research Unit, Institute for Maternal and Child Health—IRCCS “Burlo Garofolo”, 34137 Trieste, Italy

**Keywords:** Diseases, Health care

## Abstract

Patients with Type 2 Diabetes Mellitus (T2DM) are rapidly increasing in Italy due to aging, preventable risk factors, and worsening socioeconomic context. T2DM and its sequelae take a heavy toll on healthcare systems and the economy, given costly management, difficulties in coping with everyday life, and decreasing patient/worker productivity. Considering long life expectancy in Italy and a decreasing mortality rate due to T2DM, this study aims to calculate the years lived with disability (YLDs) of T2DM and its sequelae grouped into three categories: Neuropathy, Chronic Kidney Disease and No Complications, taking into consideration sex, year, and geographical location. This is the first attempt to measure YLDs from data that do not rely on self-reported diabetes diagnoses. Data come from the Italian Diabetologists Association dataset, the most comprehensive longitudinal source of national outpatient data. YLDs are obtained by multiplying the number of individuals living with a specific health condition and a disability weight which represents the magnitude of health loss associated with that particular condition. Findings show increasing YLD age-standardized rates for T2DM and its sequelae, especially Neuropathy, with the trend being stronger in the central macro-region and among men, and that 2009 marks a structural change in YLD growth rate. Systematic data collection for measuring the burden of diseases is key, among other things, to policy-making and implementation.

## Introduction

Diabetes Mellitus is a public health issue, and its pace of increase is alarming. The 10th edition of the International Diabetes Federation Atlas^1^ confirms that diabetes is an epidemic^2^, one of the fastest-growing global health emergencies of the twenty-first century with a forecast of 1.3 billion cases by 2050^3^. Diabetes elicits attention as a chronic disease associated with severe complications such as kidney failure, lower limb amputation, blindness, and cardiovascular diseases, which compromise individuals’ functional capacity, autonomy, and quality of life. Diabetes takes a high social and financial toll directly on patients and health systems, and indirectly on the economy as a result of the reduction of patients’ productivity. In particular, type 2 Diabetes Mellitus (T2DM) accounts for over 90% of diabetes worldwide^1^. Population aging, sedentary lifestyle, overweight, and obesity are considered to be the leading risk factors for the increase of T2DM incidence and prevalence. In addition, T2DM is strongly impacted by socio-economic contexts^4–7^, including urbanization^8–10^.

In 2021, the estimated global prevalence of T2DM in the adult population was nearly 10%, projected to rise to 11.3% by 2030 and to 12.2% by 2045, not to mention undiagnosed diabetes with one-in-two adults with diabetes being unaware of their condition^1,3^.

Concerning Italy, prevalence amounts to 6.4%, but other sources based on sample surveys where diabetes is self-reported provide lower estimates: 5.6% and 4.7% according to the Italian National Institute of Statistics^11^ and the PASSI surveillance system of the National Institute of Health^12^, respectively. Both ISTAT and PASSI found prevalence greater for men than women. Moreover, T2DM is a leading cause of disease burden^3,13^. Although the mortality rate decreased by more than 20% in all age groups in the last decade^11^, sedentary lifestyles, overweight, and obesity have reached alarming levels: in 2021, 33.7% of Italians were physically inactive, 34.2% of adults were overweight, and 12.0% were obese^11^.

Considering these trends, the study aims to calculate the burden of disease with regard to years lived with disability (YLDs) for T2DM and its sequelae in Italy, which has one of the longest life expectancies in the world and one of the highest population average age (46.2 years): 23.3% of Italians are ≥ 65-year-old, 7.5% are ≥ 80-year-old^14^.

We focus on YLDs, an indicator resembling physical depreciation for capital goods. When health is considered as capital^15^, like human and physical capital, each individual is born with an expected number of years of good health to be spent during their lifetime. YLDs best quantify the amount of healthy life lost due to diseases. A YLD-based approach contributes to shifting current debates on health from life expectancy to the burden of disease, which is particularly significant in high-income countries where people live longer and chronic diseases tend to prevail. This results in substantial repercussions on the individual capacity to cope with daily life, on productivity, and, ultimately, on economic growth and the financial sustainability of the health system.

To compute the YLDs, we have used data from the Associazione Medici Diabetologi (AMD) register provided by the Italian Diabetologists Association, the only existing outpatient national dataset for Italy in which diabetes is not self-reported, made available to us thanks to an exclusive collaboration research agreement. AMD data are not publicly available. This dataset can only be accessed for research purposes and by persons who have signed a specific research collaboration agreement with the Italian Diabetologists Association.

This manuscript depicts the first attempt to calculate YLDs in Italy directly from registry data in which diabetes is clinically detected according to the American Diabetes Association. Previous evidence was based on estimated prevalence from sample surveys and self-reported disease, and no national study provided information on the burden of disease. In the absence of a national diabetes registry, the AMD dataset is the only tool in existence comparable to a registry^16^. Our contribution fills a gap in the literature by calculating YLDs directly from registry data and providing information on the burden of T2DM.

Without specifically pursuing inferential or representative goals, this investigation offers a comprehensive description of disease progression as observed through the lens of patients enrolled in AMD centers.

Also, considering the country’s extraordinary regional disparities as well as the high impact of the Great Recession on its overall social and economic contexts, Italy represents a particularly interesting setting for measuring YLDs for T2DM and improving models of intervention for prevention, healthcare, work organization, and policy agendas. Italian regions differ markedly by the size and age structure of their population and by socioeconomic contexts that are mainly, though not exclusively, related to the country’s historic and persistent North-Center and South developmental gap^17^. Moreover, the 2008 global economic crisis hit Italy particularly hard due to the already long-standing sluggish economy with a high rate of structural unemployment. Because of the exorbitant public debt, fiscal policy was necessarily restrictive and required mandatory reforms on account of the Stability Pact signed to enter the European Union. The time of austerity lasted until 2018. As a consequence, access to health became more difficult and unequal, and the socio-economic context deteriorated sharply^17–19^.

The paper is organized as follows. The next section describes the data and YLDs method of calculation. Section “Results” presents the results on the burden of T2DM and its sequelae. Section “Discussion” discusses our findings and outlines strengths and limitations of this work, while Section “Conclusion” concludes with policy implications. Additional information is provided in the Supplementary Materials section.

## Data and methods

### Data source

The AMD dataset is available from the Italian Diabetologists Association, a member of the International Diabetes Federation. It is a national longitudinal dataset collected since 2004 in almost 300 centers distributed across all 21 Italian regions. The AMD dataset is the only existing national source where T2DM is clinically diagnosed according to the American Diabetes Association^20^. In the absence of a national diabetes registry, the AMD dataset is the only tool in existence comparable to a registry^16^. Data collection and methods are carried out in accordance with relevant guidelines and regulations. Records are organized in an online software system based on computerized medical records created for each patient, ensuring the standardized extraction of the information necessary to create the AMD dataset. The dataset includes information on the following: all centers affiliated with the Italian Diabetologists Association identified by a unique code and a code for the region in which it is located; all patient demographics data, including dates of disease onset; all follow-up visit information, such as clinical exams and laboratory tests that a patient has undergone. Additionally, the AMD dataset records the occurrence of sequelae associated with T2DM. All patients enrolled give their informed consent for inclusion before they participate. The AMD dataset collection has been conducted in accordance with the Declaration of Helsinki, and the protocol was approved by the Ethics Committee of AMD. Data in the AMD registry pertains to patients who do not need hospitalization, and the records pertain to on-site visits to the AMD center. Consequently, data for patients are included in the dataset for a given year if they have undertaken at least one visit. During the visit, all necessary information is collected and associated with that visit which is exclusively voluntary. Patient data are anonymized using an ID.

For the sake of simplicity, the 21 Italian regions have been mapped into three macro-regions: North (Piedmont, Lombardy, Veneto, Emilia-Romagna, Liguria, Friuli-Venezia Giulia, Trentino-Alto Adige, Valle d’Aosta), Center (Tuscany, Umbria, Marche, Lazio, Abruzzo) and South and Isles (Campania, Puglia, Calabria, Basilicata, Sicily, Sardinia).

Every step of our research adhered to the GATHER (Guidelines for Accurate and Transparent Health Estimates Reporting) statement.

### Methods

Years lived with disability (YLDs) is a measure that quantifies the number of healthy years of life lived with diseases. It is an important indicator used to assess the burden of disease, along with the years of life lost (YLLs) due to premature death. When combined, YLDs and YLLs provide the overall burden measured in terms of Disability-Adjusted Life Years (DALYs)^21–25^.

YLDs are derived by multiplying the number of individuals living with a specific health condition by a disability weight^26^. The disability weight represents the magnitude of health loss associated with that particular condition. These weights are measured on a scale ranging from 0 to 1, where 0 represents a state equivalent to full health, and 1 represents a state equivalent to death^27^.

To calculate YLDs for T2DM and its sequelae, we had to match the list of all available sequelae in the AMD dataset with the Global Burden of Disease Study classification^28^.

This includes comprehensive descriptions of health states, which are necessary to identify the corresponding disability weights. In the AMD dataset, sequelae such as blindness, neuropathy, diabetic foot, and amputation were easily identified and linked to their corresponding disability weights. For other sequelae, we extracted the relevant information on the diagnosis from 134,263,099 medical records from the AMD dataset. The only sequela that could not be traced was kidney transplant.

Aimed to monitor the trend of some of the sequelae recurring more frequently, we categorized them into three main groups (Table 1): chronic kidney disease (CKD), neuropathy (NEURO), and No-Complications. The No-Complications group consists of patients living with T2DM who have not yet developed any complications. To account for the Italian aging population, frequencies have been age-standardized using the age distribution of the average population over the 12 years considered (2005–2016).

**Table 1:** Distribution of age-standardized YLDs rates per 100,000 patients by time and type of sequela.

Year	Age-standardized rates per 100,000 patients		
	YLDs	YLDs CKD	YLDs NEURO
2005	5054.3	167.5	432.4
2006	5278.0	258.3	755.8
2007	5514.1	324.9	1023.9
2008	5651.4	363.4	1208.2
2009	5769.5	403.4	1350.0
2010	5821.5	416.3	1426.9
2011	5855.8	423.9	1483.3
2012	5914.9	465.2	1546.6
2013	6003.0	508.7	1649.9
2014	6082.7	546.9	1734.3
2015	6180.4	591.1	1813.9
2016	6219.9	536.4	1883.2
CAGR	1.74%	10.19%	13.04%

YLDs have been calculated using the following formula:

1 $${\text{YLDs }} = {\text{ P }} \times {\text{ Dw}}$$

where, P = Prevalence of the sequela, Dw = Disability Weight for the considered sequela.

In the case of comorbidity:

2 $${\text{YLDs }} = {\text{ P }} \times \, \left[ {{1 } - \, \left( {{1 } - {\text{ DwA}}} \right) \, \times \, \left( {{1 } - {\text{ DwB}}} \right)} \right]$$

where, P = Prevalence of sequela A and B, DwA = Disability Weight for A, DwB = Disability Weight for B.

YLDs were computed and shown as age-standardized rates per 100,000 patients population by sex, location, and year, for overall T2DM and two groups of sequelae, namely those recurring most frequently in the AMD dataset. For our purpose, we report merely age-standardized YLDs for 100,000 patients in the “Results” section.

To age-standardize the YLDs rates, we utilize the average age structure of the patients observed throughout the study period computing the average number of patients in each age class over the 12 years. This ensures that age differences do not confound the comparisons and provides a more accurate representation of the burden of disease across different time points. At the same time, it allows for a reduction of the effect of selecting the age structure of a reference standard population (i.e. habitants) of just one specific year in the 12 years under study.

To calculate the mean annual growth rate of YLDs across 2005–2016, we use a Compound annual growth rate (CAGR). It provides a constant smoothed annual rate of growth that, when compounded each year, is equivalent to the actual annual growth rate and allows for comparisons between different phenomena:

3 $${{\text{CAGR}}=\left(\frac{{Y}_{t}}{{Y}_{0}}\right)}^\frac{1}{t}-1$$

where Y_o_ and Y_t_ are the quantities of interest at time 0 and time *t* respectively, and *t* is the number of time periods between the two observations. Positive values indicate growth, and larger values imply a faster rate of growth.

Structural changes for the data-generating mechanism for YLDs have been tested using the Chow test. The null hypothesis was that the parameters of the linear model representing the trend of the YLDs remained constant throughout the considered period. Significant values for the test in a specific year suggest a change in the trend for that year.

Confidence intervals for YLDs have been computed assuming a Poisson distribution for the data-generating process. The significance level used was of $\alpha =0.05$.

The average duration of stay for patients in the study was assessed using Kaplan–Meier product limit estimates and restricted mean survival time (RMST). Time-to-event data were reconstructed by considering the time of each patient's last visit to a center. Observations in 2016 were treated as censored.

Analyses have been carried out using R software v. 4.2.2.

## Results

### Descriptive statistics

Our study focuses on the records of adult patients aged 15 and older with a diagnosis of T2DM capturing data over a 12-year period (from 2005 to 2016). In 2016, the AMD dataset reports 4,586,983 records referring to 524,487 patients with T2DM. The number of those enrolled has substantially increased since 2005, starting from 192,942 patients in 2005 (87,549 women and 105,393 men), and reaching 524,487 of which 228,289 are women and 296,198 are men (Table 2). The sex ratio shows a larger prevalence for men, 20.4% more in 2005, increasing over time at a compound annual growth rate (CAGR) of 0.55%, resulting in a 30% higher prevalence for men in 2016.

**Table 2:** Number of patients by sex and year.

Year	Patients			Average age (SD) years	
	F	M	Sex ratio (%)	M	F
2005	87,549	105,393	120.4	67.79 (10.91)	64.73 (10.63)
2006	105,953	127,544	120.4	68.20 (10.93)	65.21 (10.68)
2007	125,994	152,484	121	68.44 (11.06)	65.45 (10.76)
2008	144,050	175,193	121.6	68.75 (11.13)	65.79 (10.83)
2009	154,750	189,435	122.4	69.06 (11.14)	66.12 (10.86)
2010	171,912	212,249	123.5	69.28 (11.26)	66.41 (10.91)
2011	182,626	226,222	123.9	69.57 (11.30)	66.85 (10.94)
2012	195,301	244,447	125.2	69.88 (11.33)	67.17 (11.00)
2013	203,330	258,555	127.2	70.17 (11.36)	67.47 (11.01)
2014	212,353	272,045	128.1	70.45 (11.38)	67.81 (11.00)
2015	225,155	289,956	128.8	70.79 (11.39)	68.17 (11.04)
2016	228,289	296,198	129.7	71.13 (11.44)	68.53 (11.05)

Geographically, patients are distributed differently across the country, in line with the geographical distribution of the AMD centers. In 2016, the AMD centers by geographical area (see Supplementary Table S1) are mostly concentrated in the North, accounting for 56% of the total; 24% of them are in the Center, and 21% are in the South and Isles. During the whole observational period, the geographical distribution has remained stable, both for the number and the location of the centers. This results in a higher concentration of patients in the North compared to the South and Isles (Table 3).

**Table 3:** Distribution of the number of patients by geographical area and year (counts and percentages by row).

Year	Counts				Percentages by row (%)		
	Center	North	South & islands	Total	Center	North	South & islands
2005	52,859	107,813	32,270	192,942	27.4	55.9	16.7
2006	63,716	128,232	41,549	233,497	27.3	54.9	17.8
2007	73,488	153,886	51,104	278,478	26.4	55.3	18.4
2008	83,369	177,665	58,209	319,243	26.1	55.7	18.2
2009	89,344	192,315	62,526	344,185	26	55.9	18.2
2010	98,006	213,750	72,405	384,161	25.5	55.6	18.8
2011	105,924	225,319	77,605	408,848	25.9	55.1	19
2012	113,461	245,714	80,573	439,748	25.8	55.9	18.3
2013	123,040	255,204	83,641	461,885	26.6	55.3	18.1
2014	132,761	265,394	86,243	484,398	27.4	54.8	17.8
2015	145,039	282,407	87,665	515,111	28.2	54.8	17
2016	150,813	285,596	88,078	524,487	28.8	54.5	16.8

According to the geographical percentage distribution per year (row profiles), the prevalence of patients in the Center increased over time. From 2011 to 2016, it went from 25.9 to 28.8%, while those from the North remained relatively stable, from 55.1% in 2011 to 54.5% in 2016. Furthermore, the relative prevalence in the South and Isles shows a tendency to decrease going from a peak of 19% of patients in 2011 to 16.8% in 2016.

As expected, given the age-related nature of T2DM, the average age of the patients falls between 65 and 72 years, with standard deviations remaining relatively stable over time, ranging from 10.6 to 11.4 years. Men, on average, consistently exhibit older ages compared to women (Table 2).

Patients from the Center (Table 4) tend to be slightly older than those from the other two geographical areas. The differences in age among the three geographical areas appear to become less and less relevant in recent years.

**Table 4:** Average age (in years) and Standard deviations (in years) by geographical area and year.

Year	Average age (SD) in Years		
	Center	North	South & islands
2005	66.60 (10.88)	66.01 (10.83)	65.69 (10.91)
2006	67.08 (10.90)	66.48 (10.85)	66.04 (10.99)
2007	67.37 (10.96)	66.80 (10.98)	66.02 (11.02)
2008	67.73 (11.02)	67.10 (11.06)	66.34 (11.07)
2009	67.98 (11.08)	67.42 (11.10)	66.72 (11.01)
2010	68.16 (11.19)	67.69 (11.18)	67.08 (11.03)
2011	68.50 (11.23)	68.08 (11.22)	67.45 (10.99)
2012	68.77 (11.27)	68.40 (11.26)	67.73 (11.05)
2013	68.99 (11.28)	68.70 (11.29)	68.04 (11.03)
2014	69.21 (11.26)	69.04 (11.30)	68.35 (11.03)
2015	69.56 (11.23)	69.36 (11.36)	68.75 (11.00)
2016	69.91 (11.27)	69.69 (11.39)	69.15 (11.00)

#### Duration of stay in the study

Median duration for the stay in the study for all patients, by sex and geographical area (see Supplementary Table S2), is 7 years. Restricted mean survival time (RMST) estimates show an average duration of 6.21 years for all patients, with slightly longer durations for men (6.24 years for men versus 6.18 years for women). Duration also varies across geographic locations, decreasing progressively from the North (6.26 years) to the Center (6.17 years) and then to the South and Isles (6.12 years).

Table 5 displays the number of patients categorized by year and type of sequelae, both in crude form and age-standardized. To identify trends, the Compound Annual Growth Rate (CAGR) over the 12 years has been calculated.

**Table 5:** Counts of observed sequelae and standardized counts by year and type.

Frequency					Frequency standardized by age			
Year	CKD	NEURO	No-compl	ALL	Year	CKD	NEURO	No-compl
2005	13,367	6636	171,602	194,466	2005	26,167	12,727	340,652
2006	22,398	13,272	196,791	237,243	2006	36,057	21,716	322,775
2007	30,819	20,940	226,469	285,187	2007	42,084	29,054	310,791
2008	39,708	28,314	251,699	328,875	2008	47,665	34,233	301,015
2009	47,676	34,303	263,974	357,006	2009	53,010	38,254	293,056
2010	57,182	40,444	289,328	399,952	2010	57,180	40,770	287,206
2011	64,238	45,312	303,024	427,154	2011	60,243	42,752	282,843
2012	73,552	51,538	319,802	461,011	2012	63,776	44,946	278,070
2013	82,840	58,027	328,255	486,722	2013	68,186	47,960	272,086
2014	91,019	64,028	338,661	512,607	2014	70,986	50,156	268,256
2015	98,642	70,215	357,667	546,832	2015	72,698	52,108	265,824
2016	97,111	75,227	364,205	557,627	2016	69,738	54,004	266,942
CAGR	17.97%	22.43%	6.47%	9.18%	CAGR	8.51%	12.80%	− 2.01%

**Figure 1:**
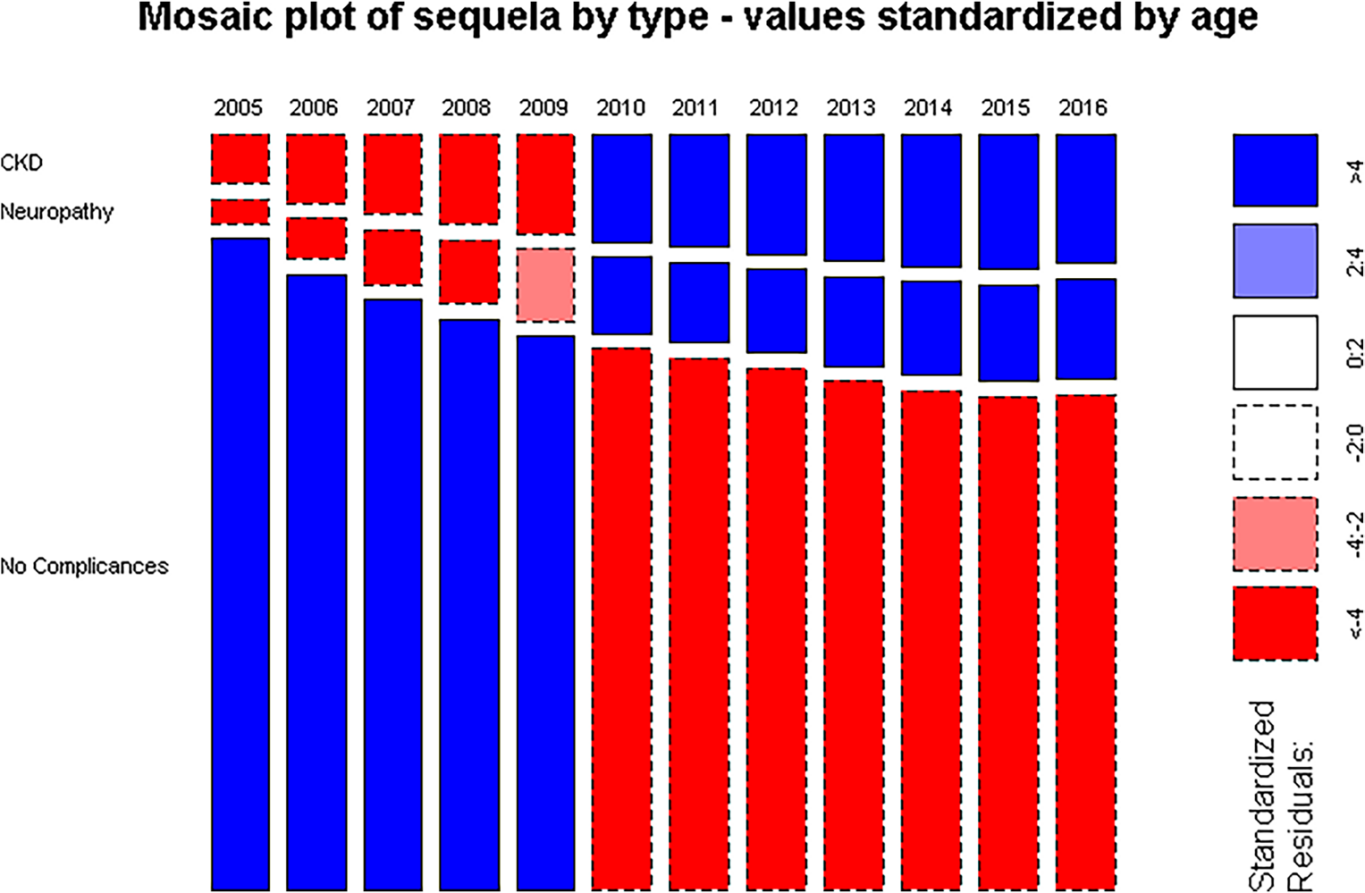
Mosaic plot of the type of sequelae over time for the observed counts standardized by age.

There is a decreasing trajectory in the evolution of standardized frequencies by age for the No-Complications group as time progresses from 2005, with a CAGR of − 2.0% per year. This is contrasted by an increase in both CKD and NEURO, with a CAGR of 8.5% and 12.8%, respectively. In detail, the mosaic plot in Fig. 1 displays the relative prevalence of the sequela groups over time.

**Figure 2:**
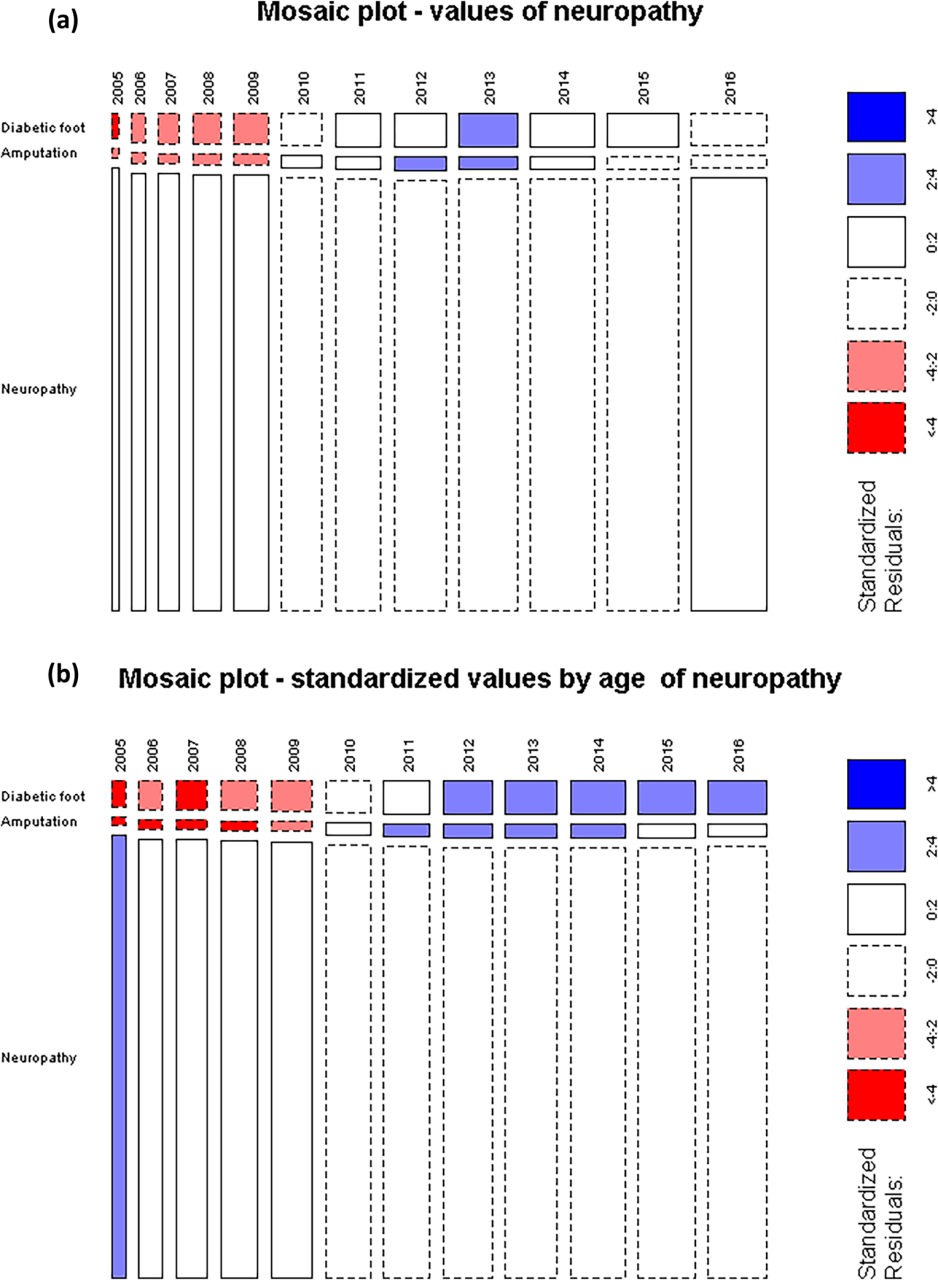
Mosaic plot for neurologic complications by time; observed counts (**a**) and age-standardized counts (**b**).

The standardized residuals, calculated under the assumption of independence between time and sequelae prevalence, reveal an increasing deviation from the expected counts for symptomatic sequelae, with larger than expected relative prevalences after 2010 for CKD and NEURO. Simultaneously, starting the same year, there is a noticeable decrease in the relative prevalence of patients with No-Complications suggesting a worsening of the seriousness of the disease over time. This implies that the relative composition of counts of sequelae per year tends to change in favor of a larger fraction of CKD and NEURO sequelae over time. Particularly, from 2011, NEURO presents the largest CAGR, with larger than expected counts for “amputation” and “diabetic foot” (Fig. 2).

### YLDs

In Italy, in 2016, for both sexes, overall age-standardized YLD rates per 100,000 were 6220, of which 536 for CKD, and 1883 for NEURO (see Supplementary Table S7). The CAGR was 1.74% for overall YLDs, 10.19%, and 13.04% for CKD and NEURO, respectively.

With regard to sex, overall YLD age-standardized rates were higher in men, though YLDs for CKD affected women slightly more than men. Considering the growth rate, between 2005 to 2016, the CAGR was higher for NEURO in women (see Table 6).

**Table 6:** Distribution of age standardized YLDs rates per 100,000 patiens by year, sex and type of sequela.

Year	Age standardized YLDs for men			Age standardized YLDs for women		
	YLDs	YLDs CKD	YLDs NEURO	YLDs	YLDs CKD	YLDs NEURO
2005	5026.6 (4887.6; 5165.6)	162.3 (137.3; 187.3)	478.3 (435.4; 521.2)	5088.6 (4948.8; 5228.4)	173.9 (148.1; 199.7)	375.4 (337.4; 413.4)
2006	5273.1 (5130.8; 5415.4)	252.4 (221.3; 283.5)	838 (781.3; 894.7)	5284.1 (5141.6; 5426.6)	265.5 (233.6; 297.4)	653.8 (603.7; 703.9)
2007	5503.2 (5357.8; 5648.6)	315.3 (280.5; 350.1)	1108.7 (1043.4; 1174)	5527.7 (5382; 5673.4)	336.8 (300.8; 372.8)	918.6 (859.2; 978)
2008	5636.4 (5489.3; 5783.5)	350.6 (313.9; 387.3)	1295.4 (1224.9; 1365.9)	5670.1 (5522.5; 5817.7)	379.2 (341; 417.4)	1100.1 (1035.1; 1165.1)
2009	5765.3 (5616.5; 5914.1)	390 (351.3; 428.7)	1445.9 (1371.4; 1520.4)	5774.7 (5625.8; 5923.6)	420.1 (379.9; 460.3)	1231 (1162.2; 1299.8)
2010	5826.2 (5676.6; 5975.8)	400 (360.8; 439.2)	1534.7 (1457.9; 1611.5)	5815.6 (5666.1; 5965.1)	436.4 (395.5; 477.3)	1293.2 (1222.7; 1363.7)
2011	5859.3 (5709.3; 6009.3)	409 (369.4; 448.6)	1587.2 (1509.1; 1665.3)	5851.5 (5701.6; 6001.4)	442.5 (401.3; 483.7)	1354.4 (1282.3; 1426.5)
2012	5931 (5780.1; 6081.9)	446 (404.6; 487.4)	1660.1 (1580.2; 1740)	5895 (5744.5; 6045.5)	489 (445.7; 532.3)	1405.9 (1332.4; 1479.4)
2013	6030.4 (5878.2; 6182.6)	494.2 (450.6; 537.8)	1775.3 (1692.7; 1857.9)	5969.1 (5817.7; 6120.5)	526.6 (481.6; 571.6)	1494.4 (1418.6; 1570.2)
2014	6120.8 (5967.5; 6274.1)	533.4 (488.1; 578.7)	1872.6 (1787.8; 1957.4)	6035.5 (5883.2; 6187.8)	563.6 (517.1; 610.1)	1562.8 (1485.3; 1640.3)
2015	6218.5 (6063.9; 6373.1)	569.8 (523; 616.6)	1959.4 (1872.6; 2046.2)	6133.2 (5979.7; 6286.7)	617.4 (568.7; 666.1)	1633.3 (1554.1; 1712.5)
2016	6264.5 (6109.4; 6419.6)	515.8 (471.3; 560.3)	2033.7 (1945.3; 2122.1)	6164.6 (6010.7; 6318.5)	562.1 (515.6; 608.6)	1696.6 (1615.9; 1777.3)
CAGR	1.85%	10.11%	12.82%	1.61%	10.27%	13.39%

Geographically, due to the distribution of AMD centers, the North and the Center account for almost 80% of centers (see Supplementary Table S1). In view of this, we report and describe results for the two geographical areas using the South and Isles macro-region as a reference value. The values for the South and Isles are not shown. The values depict a burden of disease variation across Italy: The Center shows the overall highest age standardized YLDs rates and the highest CAGR between 2005 and 2016. Nevertheless, in 2016, YLDs for CKD were greatest in the North.

Chow test performed on the time series of the age-standardized YLD rates of T2DM and its sequelae suggests a structural change for the years 2008 (F = 62.67, p = 0.000013) and 2009 (F = 36.58, p = 0.000094) (see Supplementary Table S3) when the growing trend of age-standardized YLD rates started to reduce.

## Discussion

T2DM and its sequelae have a major impact on the Italian health system and society. The initial indication is shown in the mosaic plot. Over the years, there has been a consistent decline in the proportion of patients without complications, coupled with an increase in patients affected by CKD and neurological disorders, highlighting a growing severity of the disease due to the increase in the proportion of patients with comorbidities. This is further stressed by the larger increase of the quota due to NEURO, which in 2016 nearly equals the one due to CKD. Together, they account for a proportion of patients which is roughly half of those with no complications (Fig. 1). Our results are entirely consistent with findings in the literature, particularly those that have captured the global trends in T2DM complications in high-income countries^29^.

Concerning YLDs, we see an overall increasing trend, with NEURO having a pronounced higher CAGR. The observed CAGR indicates the dynamic nature of these health outcomes confirming what has been reported in the literature about an increasing trend of the burden of T2DM and its gradual worsening^3,30^ and, reaffirms that T2DM is a major cause of disability^3^. YLDs are higher for men, verifying what is well-known in the literature about sex differences in risk, pathophysiology, and complications^31^.

Geographically, our findings show that patients in the North have a longer duration of stay in the AMD center compared to those in the Center and the South and Isles. This might be attributed to better awareness, lower deprivation, and greater availability of healthcare services, factors that prevail in the northern regions relative to the rest of the country^32^. These findings are consistent with the literature on the effects of socioeconomic factors on T2DM^19,33,34^. The different trends in the geographical distribution of patients may support the hypothesis of medical commuting, a well-known phenomenon that particularly affects patients from the South and Isles^11^.

Both overall and NEURO YLDs are higher in the Center while CKD YLDs are bigger in the North. The Center is also dominant for the CAGR that is 10.29% and 14.58% for CKD and NEURO respectively (Table 7). Italy suffers from a historical and persistent developmental gap between the North with respect to other geographical areas for social and economic deprivation, inefficient public services, environmental damage, unemployment, and even crime, all factors pertaining to the socioeconomic context that have proven to affect health^19^.

**Table 7:** Distribution of age standardized YLDs rates per 100,000 patiens by year, geographical area and type of sequela.

Year	Age standardized YLDs for Central region			Age standardized YLDs for the North region		
	YLDs	YLDs CKD	YLDs NEURO	YLDs	YLDs CKD	YLDs NEURO
2005	5071 (4931.4; 5210.6)	144.4 (120.8; 168)	513.8 (469.4; 558.2)	5116.6 (4976.4; 5256.8)	247.6 (216.8; 278.4)	493 (449.5; 536.5)
2006	5453.9 (5309.2; 5598.6)	264.9 (233.; 296.8)	1001.3 (939.3; 1063.3)	5276 (5133.6; 5418.4)	323.7 (288.4; 359)	743.8 (690.3; 797.3)
2007	5779.2 (5630.2; 5928.2)	314 (279.3; 348.7)	1408.1 (1334.6; 1481.6)	5433.8 (5289.3; 5578.3)	380.7 (342.5; 418.9)	909.8 (850.7; 968.9)
2008	5983.1 (5831.5; 6134.7)	333.9 (298.1; 369.7)	1701.9 (1621; 1782.8)	5536.2 (5390.4; 5682)	439.3 (398.2; 480.4)	1009.6 (947.3; 1071.9)
2009	6122.4 (5969; 6275.8)	355.9 (318.9; 392.9)	1872.9 (1788.1; 1957.7)	5658.4 (5511; 5805.8)	497.5 (453.8; 541.2)	1135.3 (1069.3; 1201.3)
2010	6197.4 (6043.1; 6351.7)	357.1 (320.1; 394.1)	1961.7 (1874.9; 2048.5)	5734.9 (5586.5; 5883.3)	505.9 (461.8; 550)	1233.7 (1164.9; 1302.5)
2011	6269.3 (6114.1; 6424.5)	377.1 (339; 415.2)	2025.9 (1937.7; 2114.1)	5745.2 (5596.6; 5893.8)	484.2 (441.1; 527.3)	1263.3 (1193.6; 1333)
2012	6330.2 (6174.3; 6486.1)	406.5 (367; 446)	2122.2 (2031.9; 2212.5)	5810.9 (5661.5; 5960.3)	539.6 (494.1; 585.1)	1293 (1222.5; 1363.5)
2013	6465 (6307.4; 6622.6)	444 (402.7; 485.3)	2288.3 (2194.5; 2382.1)	5896.5 (5746; 6047)	592.7 (545; 640.4)	1378.5 (1305.7; 1451.3)
2014	6555.9 (6397.2; 6714.6)	467.4 (425; 509.8)	2428.8 (2332.2; 2525.4)	5989.9 (5838.2; 6141.6)	646.7 (596.9; 696.5)	1444.8 (1370.3; 1519.3)
2015	6641.3 (6481.6; 6801)	468 (425.6; 510.4)	2523.5 (2425; 2622)	6122 (5968.6; 6275.4)	719.8 (667.2; 772.4)	1546.3 (1469.2; 1623.4)
2016	6729.2 (6568.4; 6890)	467.8 (425.4; 510.2)	2631.5 (2531; 2732)	6112.5 (5959.3; 6265.7)	600.8 (552.8; 648.8)	1607.4 (1528.8; 1686)
CAGR	2.39%	10.29%	14.58%	1.49%	7.67%	10.34%

The structural change observed since 2009 marks a significant turning point. Notably, there has been a more pronounced reduction in patients without complications starting from 2010, while the burden of the disease increases more in men compared to women. That could suggest a potential simultaneous effect of deteriorating lifestyles and difficulties in accessing healthcare. Since the Great Recession of 2008, in fact, the Italian healthcare sector has been hit with the consequence of a strong supply reduction. The major effects were the lengthening of waiting lists, increased difficulties in accessing treatment, and foregoing treatments due to economic reasons^18^. Furthermore, from a general point of view, the 2008 crisis reduced incomes, mainly of the middle class, and increased poverty and deprivation^32^ which has led to an overall deterioration of the socio-economic context. Our results confirm what has already been reported in the literature about the effects of the Great Recession on health, particularly on T2DM^35–37^.

This study has several strengths that contribute to its significance. Firstly, it represents the first-ever investigation conducted in Italy on such extensive data, providing novel insights into the burden of T2DM and its sequelae. Secondly, the use of the exclusive AMD dataset enables us to calculate YLDs directly from clinical data, offering valuable contributions to the understanding of the impact of the disease. Unlike other available national sources in Italy, which rely on self-reported diagnoses through sample surveys, the AMD dataset provides longitudinal and clinically diagnosed information on T2DM and its sequelae. Finally, this study considers the deterioration of the socio-economic context in the Italian macro-regions by verifying the presence of a structural change in the observed series coinciding precisely with the years of the Great Recession.

Furthermore, the metrics we used have the unique ability to measure the burden showing how the severity of the disease and its sequelae are a great cause of concern. This constitutes a precious mark that may not be evident using other indicators. Our findings point to an increasing burden on the Italian healthcare systems, society, and economy, given the high cost of managing T2DM and its sequelae, the declined capability to cope with everyday life, and the reduction of patient/worker productivity.

However, this study has certain limitations. The AMD dataset does not uniformly cover all of Italy’s geographical areas and all T2DM patients. In Italy, patients with T2DM have care provided in different settings: a part is in the charge of general practitioners (GPs) while those with severe complications are in hospitals. The remaining patients, who account for 40% of the whole population, are followed by AMD centers, either exclusively or in integrated management with the GP. Patients treated by GPs are mainly elderly individuals with less severe forms of the disease, sometimes in socio-economic deprivation^38^, while those in hospitals are in more severe conditions and most of them in socio-economic deprivation^39^.

This investigation cannot capture conditions that require hospitalization (registered by hospital discharge form extensively). In addition, it must be emphasized that the study relies exclusively on administrative data and has an observational nature, which may introduce biases and limit the establishment of causal relationships.

Considering this, the first challenge is for the healthcare system to build and guarantee access to better healthcare services. To this end, a data-driven approach along with a systematic data collection system are required to drive actions at the various levels including at the policy level, as per the Lancet Commission on Diabetes’ recommendation^40^.

## Conclusion

This study aims to calculate the burden of disease—specifically, years lived with disability—due to T2DM and its sequelae in Italy, taking into consideration sex, year, and geographical location. Italy bears attention because although the mortality rate for T2DM has decreased, sedentary lifestyles, overweight, and obesity have reached alarming levels and, in the last years, the socio-economic context has deteriorated.

Our investigation represents the first attempt to measure YLDs in Italy directly from a dataset in which the diagnosis of diabetes is not self-reported. We use data from the Italian Diabetologists Association (AMD), the owner of the most comprehensive source of national outpatient data on diabetes in Italy. Considering that Italy has one of the longest life expectancies in the world and one of the highest population average ages, we concentrate on age-standardized YLDs for T2DM and its sequelae grouped into three categories. The results show an emergency issue: the worrying signal is represented by the growth of age-standardized YLD rates, revealing a progressive worsening in the disease and its sequelae between 2005 and 2016, with a structural change point in 2009, especially for men who live in the Center of Italy.

Our results constitute an important contribution by providing insights into an issue never investigated in Italy. The implication of our findings emphasizes the need to address more analyses on the burden of disease that better demostrate the dimension of a toll on the Italian healthcare system, society, and economy.

Given the high cost of managing T2DM and its sequelae, the reduction of the capability to cope with everyday life, and patient/worker productivity, our results emphasize the urgency of preventive strategy in various settings. This is a necessary condition to help contain the emerging epidemic of complications of diabetes. This study stresses the YLDs as a part of the avoidable burden.

Future developments may involve using the updated AMD dataset to detect the effect the COVID pandemic may have had on the access of patients to AMD centers, as well as the estimation of the economic burden of T2DM and its sequelae.

### Supplementary Information

Supplementary Tables.

## Data Availability

The data that support the findings of this study are available from AMD, but restrictions apply to the availability of these data, which were used under license for the current study, and so are not publicly available. Data are however available from the authors upon reasonable request and with permission from AMD.
