# Supplementary material for: Measures of type 2 diabetes burden in Italy assessed using the AMD dataset over a twelve year span across the Great Recession
Source: Sci Rep. 2024 Feb 28;14:4901. doi: 10.1038/s41598-024-54989-8 (PMC10901812; doi:10.1038/s41598-024-54989-8)
Supplement: Supplementary file 1 — Supplementary Tables. [file 41598_2024_54989_MOESM1_ESM.pdf]

Measures of type 2 Diabetes burden in Italy assessed using the AMD dataset over a twelve year span across the Great Recession

Cristiana Abbafati[1†], Luciano Nieddu[2†], Gianluca Superti[3], Lorenzo Monasta[4]
[1*]Department of Juridical and Economic Studies, Sapienza University of Rome, P.za A. Moro 5, Rome, 00185, Italy .
[2]Department of Economics, UNINT University for International Studies, Via C. Colombo, 200, Rome, 00147, Italy .
[3]Department of Statistics, Sapienza University of Rome, P.za A. Moro, 5, Rome, 00185, Italy .
[4]Clinical Epidemiology and Public Health Research Unit, Institute for Maternal and Child Health—IRCCS "Burlo Garofolo", Trieste, 34137, Italy .

*Corresponding author(s). E-mail(s): cristiana.abbafati@uniroma1.it;
Contributing authors: l.nieddu@unint.eu; g.superti@uniroma1.it; lorenzo.monasta@burlo.trieste.it;
†These authors contributed equally to this work

Supplementary Material

Geographical area/Year	2005	2006	2007	2008	2009	2010	2011	2013	2014	2015	2016
North	152	153	153	153	153	153	155	155	155	155	155
Center	64	65	65	66	66	66	66	66	66	66	66
South and Isles	56	57	57	57	57	57	57	57	57	57	57

Table S1: number of AMD centers by geographical location and year

strata	records	events	RMST	se(RMST).	median
ALL	1,058,015	533,528	6.21196	0.00462	7
sex=F	465,700	237,411	6.18128	0.00695	7
sex=M	592,315	296,117	6.23629	0.00619	7
area=Center	298,119	147,306	6.16942	0.00884	7
area=North	577,903	292,307	6.26432	0.00698	7
area=South&Isles	181,993	93,915	6.11545	0.01120	7

Table S2: restricted mean survival time (in years) and median duration for subjects in the study for the entire dataset, by sex and geographical area

year	YLDs		YLDsCKD		YLDsNEURO	
	F	p-value	F	p-value	F	p-value
2008	62.67	0.00001296	5.67	0.02931294	116.43	0.00000122
2009	36.58	0.00009444	4.90	0.04085830	43.23	0.00005146
2010	13.28	0.00287092	2.81	0.11923951	15.78	0.00167381
2011	4.74	0.04396560	1.39	0.30405201	6.58	0.02046689
2012	2.08	0.18698970	1.45	0.28984390	3.12	0.09951521
2013	1.15	0.36372540	2.04	0.19238640	1.85	0.21903240

Table S3: Chow test results for structural breaks (F values and p-values) for YLDs, YLDs CKD, and YLDs NEURO. All values are standardized by age.

Year	Rates per 100000 patients		
	YLDs	YLDs CKD	YLDs NEURO
2005	5036.0	143.9	441.9
2006	5257.4	242.0	752.2
2007	5491.0	306.8	1015.7
2008	5627.3	342.0	1201.5
2009	5747.2	385.9	1343.8
2010	5795.7	406.3	1405.5
2011	5839.9	422.8	1465
2012	5915.2	471.5	1539.9
2013	6007.7	516.9	1649.3
2014	6090.6	560.0	1739.7
2015	6187.5	604.7	1821.8
2016	6258.6	557.7	1923.9
CAGR	1.83%	11.95%	13.04%

Table S4: Distribution of the overall YLD rates per 100000 patients by time and type of sequela.

Year	YLDs rates for men			YLDs rates for women		
	YLDs	YLDs CKD	YLDs NEURO	YLDs	YLDs CKD	YLDs NEURO
2005	5003.72	137.986	490.49	5074.5	150.894	383.979
2006	5250.7	236.291	836.846	5265.51	248.717	651.164
2007	5481.22	298.701	1102.18	5502.71	316.554	911.909
2008	5614.04	331.401	1287.83	5643.39	354.8	1097.47
2009	5745.01	376.059	1437.54	5749.91	397.948	1229.98
2010	5800.07	390.275	1510.7	5790.37	425.771	1277.02
2011	5842.56	407.009	1567.92	5836.71	442.222	1338.66
2012	5928.9	451.116	1651.83	5898.1	496.683	1401.01
2013	6031.8	501.29	1771.73	5977.24	536.463	1495.03
2014	6125.6	546.446	1874.74	6046.27	577.27	1568.42
2015	6221.17	582.973	1963.92	6144.58	632.499	1640.23
2016	6300.32	536.092	2073.28	6204.96	585.518	1731.55
CAGR	1.94%	11.97%	12.76%	1.69%	11.96%	13.37%

Table S5: YLDs rates per 100000 patients by sex, year, and type of sequela.

Year	YLDs rates for the Central region			YLDs rates for the North region		
	YLDs	YLDs CKD	YLDs NEURO	YLDs	YLDs CKD	YLDs NEURO
2005	5112.94	131.191	594.85	5060.79	207.271	461.459
2006	5484.28	265.031	1076.34	5223.3	296.853	700.53
2007	5816.25	320.805	1471.88	5388.85	347.534	887.699
2008	6002.56	336.805	1739.97	5500.61	397.188	1010.95
2009	6126.23	360.467	1888.14	5630.32	461.202	1141.24
2010	6198.15	359.742	1967.59	5703.91	487.721	1214.08
2011	6267.93	380.702	2027.27	5730.42	483.296	1242.37
2012	6341.75	411.173	2136.72	5813.17	545.411	1284.75
2013	6443.51	442.28	2263.41	5912.77	603.337	1387.59
2014	6485.27	461.029	2354.34	6026.42	666.85	1476.6
2015	6502.67	453.599	2374.99	6179.97	748.062	1600.73
2016	6628.3	455.137	2524.02	6194.87	642.557	1679.41
CAGR	2.19%	10.92%	12.80%	1.70%	9.89%	11.37%

Table S6: YLDs rates per 100000 patients by geographic region, year, and type of sequela.

GROUP	Sequela	DW
CKD	Albuminuria with preserved GFR due to T2DM	0
CKD	End-stage renal disease on dialysis due to T2DM	0.571
CKD	Stage III chronic kidney disease and mild anemia due to T2DM	0.004
CKD	Stage III chronic kidney disease and moderate anemia due to T2DM	0.052
CKD	Stage III chronic kidney disease and severe anemia due to T2DM	0.149
CKD	Stage III chronic kidney disease without anemia due to T2DM	0
CKD	Stage IV chronic kidney disease untreated and mild anemia due to T2DM	0.107584
CKD	Stage IV chronic kidney disease untreated and moderate anemia due to T2DM	0.150592
CKD	Stage IV chronic kidney disease untreated and severe anemia due to T2DM	0.237504
CKD	Stage IV chronic kidney disease untreated without anemia due to T2DM	0.104
CKD	Stage V chronic kidney disease untreated and mild anemia due to T2DM	0.570724
CKD	Stage V chronic kidney disease untreated and moderate anemia due to T2DM	0.591412
CKD	Stage V chronic kidney disease untreated and severe anemia due to T2DM	0.633219
CKD	Stage V chronic kidney disease untreated without anemia due to T2DM	0.569
NEURO	Diabetic foot due to NEURO due to T2DM	0.15
NEURO	Diabetic NEURO and amputation with treatment due to T2DM	0.17
NEURO	Diabetic NEURO due to T2DM	0.133
NO-COMPLICATIONS	Uncomplicated T2DM	0.049

Table S7: Grouping of sequelae and corresponding Disability Weights
